# Role of plasminogen activated inhibitor-1 in the pathogenesis of anticoagulant related nephropathy

**DOI:** 10.3389/fneph.2024.1406655

**Published:** 2024-06-28

**Authors:** Ajay Medipally, Min Xiao, Laura Biederman, Alana Dasgupta, Anjali A. Satoskar, Samir Parikh, Iouri Ivanov, Galina Mikhalina, Sergey V. Brodsky

**Affiliations:** ^1^ Department of Pathology, The Ohio State University Wexner Medical Center, Columbus, OH, United States; ^2^ Department of Pathology, Nationwide Children’s Hospital, Columbus, OH, United States; ^3^ Department of Medicine, The Ohio State University Wexner Medical Center, Columbus, OH, United States; ^4^ Medicine, Rochester Regional Health Nephrology, Rochester, NY, United States

**Keywords:** anticoagulant related nephropathy, experimental model, plasminogen activator inhibitor, oxidative stress, acute kidney injury

## Abstract

Anticoagulant related nephropathy (ARN) is the result of glomerular hemorrhage in patients on systemic anticoagulation therapy or underlying coagulopathy. Red blood cells (RBC) that passed through the glomerular filtration barrier form RBC casts in the tubules, increase oxidative stress and result in acute tubular necrosis (ATN). The mechanisms of ARN still not completely discovered. Plasminogen activator inhibitor-1 (PAI-1) plays a significant role in the maintenance of coagulation homeostasis. We developed an animal model to study ARN in 5/6 nephrectomy (5/6NE) rats. The aim of this study was to elucidate the role of PAI-1 in the ARN pathogenesis. 5/6NE rats were treated per os with warfarin (0.75 mg/kg/day) or dabigatran (150 mg/kg/day) alone or in combination with PAI-1 antagonist TM5441 (2.5, 5.0 and 10 mg/kg/day). TM5441 in a dose dependent manner ameliorated anticoagulant-induced increase in serum creatinine in 5/6NE rats. Anticoagulant-associated increase in hematuria was no affected by TM5441. The levels of reactive oxygen species (ROS) in the kidneys were in a dose-dependent manner decreased in 5/6NE rats treated with an anticoagulant and TM5441. Our data demonstrates that PAI-1 may reduce ARN by decreasing ROS in the kidneys. Glomerular hemorrhage is not affected by anti-PAI-1 treatment. These findings indicate that while symptoms of ARN can be reduced by PAI-1 inhibition, the main pathogenesis of ARN – glomerular hemorrhage – cannot be prevented.

## Introduction

Anticoagulant related nephropathy (ARN) is a complication of anticoagulation therapy that results in acute kidney injury (AKI) in susceptible patients (1–3). Systemic anticoagulants, such as vitamin K antagonists (warfarin) or direct oral anticoagulants (such as dabigatran and apixaban) may result in ARN, but AKI may also develop in patients receiving heparin or antiplatelet medications (4). Unfortunately, the outcome of ARN is not favorable; in over two-thirds of the patients with ARN, kidney function did not recover one year after the episode of ARN (5). The mechanisms of kidney injury in ARN are not clear. We had developed an animal model to study ARN. 5/6 nephrectomy rats develop AKI when they are treated with high doses of vitamin K antagonists or dabigatran. These animals have increased hematuria, RBC casts in the kidney, and ATN (2, 3, 6). Both warfarin and dabigatran increase blood pressure in 5/6 nephrectomy rats (7). Modification of the glomerular filtration rate (GFR) by drugs did not change the severity of the ARN in 5/6 nephrectomy rats (8).

The coagulation system and the plasminogen system maintain coagulation homeostasis in mammals. The coagulation system controls hemorrhage by conversion of the soluble fibrinogen to an insoluble fibrin polymer and fibrin clots. The plasminogen system controls formation of the protease plasmin and maintains the patency of vasculature by degrading fibrin thrombi. Two main serine proteases, urokinase plasminogen activator (uPA) and tissue plasminogen activator (tPA), convert plasminogen to plasmin. Natural regulators of uPA and tPA activity are plasminogen activator inhibitors (PAI) (9). PAI are members of the family of serine protease inhibitors (SERPINs), a family of 36 proteins in humans (10). While PAI-1 normally is not produced in healthy kidneys, PAI-1 synthesis by both resident and intrarenal inflammatory cells occurs in several acute and chronic kidney diseases (11). Physiological glomerular filtration barrier (GFB) is composed of a fenestrated endothelium, the glomerular basement membrane (GBM), and the podocytes. In the podocyte-specific injury model of NEP25/LMB2 mice, there is an increase of glomerular PAI-1 mRNA and endothelial PAI-1 protein expression (12). Experimental data indicate that the glomerular expression of PAI-1 is associated with injury and inflammation, and PAI-1 promotes podocyte injury (13). Inhibition of PAI-1 ameliorates proteinuria in experimental animals (12).

The aim of the current study is to investigate the role of PAI-1 in the pathogenesis of ARN based on the 5/6 nephrectomy rat model.

## Materials and methods

This study was approved by the Institutional Animal Care and Use Committees (IACUC) at the Ohio State University.

Male Sprague Dawley rats (120–140 gm, the Charles River Laboratories, Wilmington, MA) were used for the studies. The 5/6 nephrectomy was performed under isoflurane/oxygen (1:5) anesthesia., Nephrectomy of the right kidney and resection of two thirds of the left kidney were performed simultaneously, as described previously (6). Hemostasis was achieved by hemostatic sponges (Quick clot; Z-medica Corporation, Wallingford, CT). The middle laparotomy incision was closed with a 4.0 proline, and the animals were kept at 12h/12h light/dark cycle on the standard rodent diet with free access to water.

All treatments were given at 3–4 weeks after the ablative surgery. The study groups included male 5/6NE rats only in order to avoid sexual-cycle related hormonal effects on the kidney function. The following groups were studied (n=7 in each group): 1) 5/6NE rats treated with 150 mg/kg/day (gavage, per os) dabigatran (Millipore Sigma, St. Loius, MO, cat # 211914–51-1) alone (3); 2) 5/6NE rats treated with 150 mg/kg/day (gavage, per os) dabigatran and PAI-1 inhibitor TM5441 (Millipore Sigma, St. Loius, MO, cat # 1190221–43-2) (gavage, per os, 3 doses of 10 mg/kg/day, 5 mg/kg/day and 2.5 mg/kg/day) (14, 15); 3) 5/6NE rats treated with 0.75 mg/kg/day warfarin (Millipore Sigma, St. Loius, MO, cat # 129–06-6) (gavage, per os) alone (6); 4) 5/6NE rats treated with 0.75 mg/kg/day warfarin (gavage, per os) and PAI-1 inhibitor TM5441 (gavage, per os, 3 doses of 10 mg/kg/day, 5 mg/kg/day and 2.5 mg/kg/day) (14, 15); 5) PAI-1 inhibitor TM5441 alone (gavage, per os, 3 doses of 10 mg/kg/day, 5 mg/kg/day and 2.5 mg/kg/day) (14, 15); 6) 5/6NE rats treated with vehicle were used as control (n=5). The dosage of the anticoagulants was used based on our previous studies (3, 6), the dosage of PAI-1 inhibitor TM5441 was used based on the literature data (14, 15). Three different doses were used to generate dose-response curves.

Hematuria was measured by Siemens Multistix 5 (Siemens Healthcare Diagnostics Inc, Tarrytown, NY) daily (6), and recorded in a semiquantitative scale from 0 to 3, where score 0 is absent, 1+ is trace, 2+ is moderate, and 3+ is large. Serum creatinine was measured daily in the tail blood; the samples (100 mcl) were collected via a tail puncture, as (6). At the end of the study, the animals were sacrificed, and the remnant kidney was cut longitudinally into two halves for histology and other studies. Histology of the kidney was blindly evaluated by renal pathologists (SB, LB, AS) on 2–3 mcm sections of paraffin-embedded tissue stained with hematoxylin and eosin.

Serum creatinine was measured based on the Jaffe reaction using a creatinine reagent assay kit (Raichem, San Marcos, CA) according to the manufacturer protocol (6). In brief, 10 mcl of serum was mixed with 200 mcl of working reagent at 37°C in a 96-well plate, and the absorbance was read at 510 nm at 60 sec and 120 sec on a microplate reader (Molecular Devices, Sunnyvale, CA).

Prothrombin time (PT) was measured using Electra 750 coagulation analyzer (Medical Laboratory Automation, Pleasantville, NY) according to the manufacturer protocol. Blood was mixed with 3.8% sodium citrate in a ratio of 9:1 and centrifuged at 1000 RCF for 15 minutes. Then 0.1 ml of plasma was placed in the incubation station for 3 minutes, and 0.2ml of warm thromboplastin was added and clotting time was recorded (2). We used a “surrogate” INR (sINR) by comparing PT before and after the treatment, as described in prior studies (2). The average PT in 50 control 5/6 nephrectomy rats was used as the normal PT time (21.44 sec).

Activated partial thromboplastin time (aPTT) was measured by using a Fisher Scientific Thromboscreen 200 Hemostasis Analyzer (Fisher Scientific, Middletown, VA) based on the manufacturer protocol (3). Briefly, tail blood was collected into a tube containing 3.8% sodium citrate in a ratio of 9:1. The blood was centrifuged at 3500 RPM for 10 min., 20 μl of plasma was placed in the incubation station with 20 μl of the aPTT reagent (Fisher scientific, Middletown, VA), and 20 μl of 0.025M pre-heated calcium chloride was added 3 minutes later. Clotting time was recorded in seconds.

Blood pressure was measured by a tail cuff using a blood pressure monitor (IITC Life Sciences Inc. Woodland Hills, CA) (7). The systolic, diastolic, and mean blood pressures were determined using the Blood Pressure Data Acquisition Software (IITC Life Sciences Inc. Version 1.35).

The levels of oxidized proteins were analyzed in the cortex of the kidneys by the Protein Carbonyl Assay (Cayman Chemical Company, MI) (16, 17) based on the manufacturer protocol. Briefly, 100 mg of the renal cortex was homogenized in ice-cold 2-(N -morpholino)ethanesulfonic acid (MES) buffer (pH 6.7) and centrifuged at 10,000xg for 15 min at 4°C. To the supernatant, 0.8 ml of 2,4-dinitrophenylhydrazine (DNPH) was added. For control samples, 0.8ml of 2.5 M HCL was added. Samples were incubated in the dark at room temperature for 1 hour with vortexing every 15 minutes. The samples were precipitated first with 1 mL of 20% trichloroacetic acid (TCA) followed by 10% TCA and then centrifuged at 10,000 g for 10 min. The pellet was washed thrice with 1 mL of ethanol-ethyl acetate (1:1; v/v) to remove free DNPH reagent and centrifuged for 10 min at 10,000 g. The protein pellet was resuspended in 0.5 mL of guanidine hydrochloride by vortexing. Samples were centrifuged at 10,000 x g for 10 mins at 4°C. The concentration of DNPH in the supernatant was determined spectrophotometrically at 370 nm (Versa Max, Molecular Devices), and the molar absorption coefficient of 22, 000 M^-1^ cm^-1^ was used to quantify the levels of protein carbonyls. Protein concentration was determined in the samples by the equation: Protein Carbonyl (nmol/ml) = [(CA)/(*0.011µM-1](500/200 µl), where CA is the corrected absorbance of the samples (16).

### Statistical analysis

Results are presented as mean ± standard deviation (SD) if not otherwise specified. Differences between 2 groups were analyzed by the two-paired *t*-test or two-way ANOVA with Tukey post-test, where applicable. GraphPad, version Prism5, GraphPad software (Boston, MA) was used to analyze data. Origin, version 2023b. OriginLab Corporation (Northampton, MA) was used to plot the graphs.

## Results

### PAI-1 inhibitor TM5441 does not affect coagulation in 5/6 nephrectomy rats

**Figure 1:**
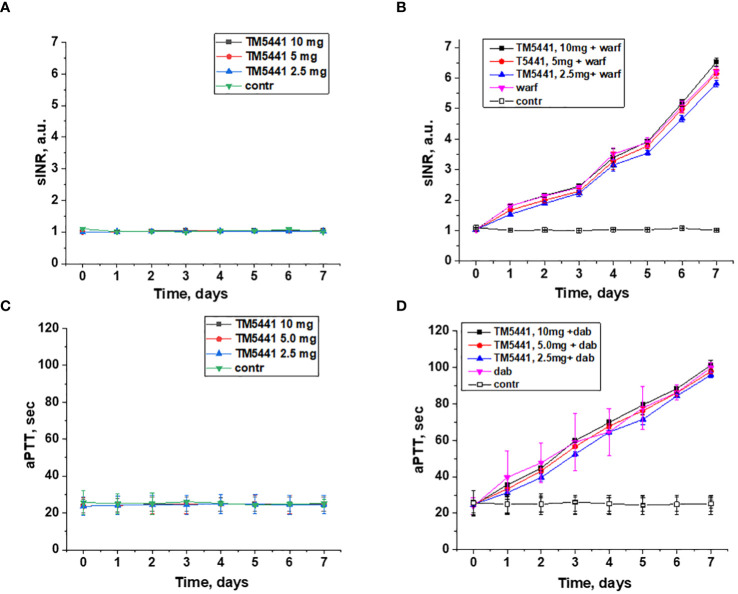
Changes in the coagulation parameters in 5/6 nephrectomy rats treated with warfarin, dabigatran and TM5441. **(A)** changes in PT time (shown as sINR) in 5/6 nephrectomy rats treated with TM5441 2.5 mg/kg/day, 5.0 mg/kg/day and 10 mg/kg/day. Vehicle treated 5/6 nephrectomy rats were used as control. **(B)** changes in PT time (shown as sINR) in 5/6 nephrectomy rats treated with 0.75 mg/kg/day warfarin (warf) and TM5441 2.5 mg/kg/day, 5.0 mg/kg/day and 10 mg/kg/day. Vehicle treated 5/6 nephrectomy rats were used as control. **(C)** changes in aPTT time in 5/6 nephrectomy rats treated with TM5441 2.5 mg/kg/day, 5.0 mg/kg/day and 10 mg/kg/day. Vehicle treated 5/6 nephrectomy rats were used as control. **(D)** changes in aPTT time in 5/6 nephrectomy rats treated with 150 mg/kg/day dabigatran (dab) and TM5441 2.5 mg/kg/day, 5.0 mg/kg/day and 10 mg/kg/day. Vehicle treated 5/6 nephrectomy rats were used as control.

Treatment with TM5441 alone did not affect either PT (calculated as sINR) or aPTT in 5/6 nephrectomy rats in any dose (2.5, 5.0, or 10.0 mg/kd/day) (**Figures 1A, C**). Combined treatment with TM5441 and 0.75 mg/kg/day warfarin did not change sINR in 5/6 nephrectomy rats as compared to the 5/6 nephrectomy rats treated with 0.75 mg/kg/day warfarin alone (**Figure 1B**). Similarly, aPTT was not changed in 5/6 nephrectomy rats treated with 150 mg/kg/day dabigatran and TM5441 in 3 different doses (2.5, 5.0, or 10.0 mg/kd/day) as compared to 5/6 nephrectomy rats treated with 150 mg/kg/day dabigatran alone (**Figure 1D**).

### Effects of PAI-1 inhibitor TM5441 on blood pressure in 5/6 nephrectomy rats

**Figure 2:**
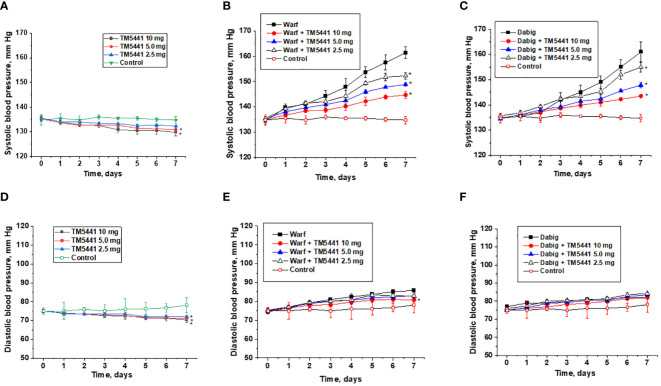
Changes in the blood pressure in 5/6 nephrectomy rats treated with warfarin, dabigatran and TM5441. **(A)** changes in the systolic blood pressure in 5/6 nephrectomy rats treated with TM5441 2.5 mg/kg/day, 5.0 mg/kg/day and 10 mg/kg/day. Vehicle treated 5/6 nephrectomy rats were used as control (contr). * - p<0.05 as compared to control. **(B)** changes in the systolic blood pressure in 5/6 nephrectomy rats treated with 0.75 mg/kg/day warfarin (warf) and TM5441 2.5 mg/kg/day, 5.0 mg/kg/day and 10 mg/kg/day. Vehicle treated 5/6 nephrectomy rats were used as control (contr). * - p<0.05 as compared to warfarin alone. **(C)** changes in systolic blood pressure in 5/6 nephrectomy rats treated with 150 mg/kg/day dabigatran (dab) and TM5441 2.5 mg/kg/day, 5.0 mg/kg/day and 10 mg/kg/day. Vehicle treated 5/6 nephrectomy rats were used as control (contr). * - p<0.05 as compared to dabigatran alone **(D)** – changes in diastolic blood pressure in 5/6 nephrectomy rats treated with TM5441 2.5 mg/kg/day, 5.0 mg/kg/day and 10 mg/kg/day. Vehicle treated 5/6 nephrectomy rats were used as control (contr). * - p<0.05 as compared to control. **(E)** changes in diastolic blood pressure in 5/6 nephrectomy rats treated with 0.75 mg/kg/day warfarin (warf) and TM5441 2.5 mg/kg/day, 5.0 mg/kg/day and 10 mg/kg/day. Vehicle treated 5/6 nephrectomy rats were used as control (contr). * - p<0.05 as compared to warfarin alone **(F)** – changes in diastolic blood pressure in 5/6 nephrectomy rats treated with 150 mg/kg/day dabigatran (dab) and TM5441 2.5 mg/kg/day, 5.0 mg/kg/day and 10 mg/kg/day. Vehicle treated 5/6 nephrectomy rats were used as control (contr).

Both warfarin and dabigatran increase systolic blood pressure in 5/6 nephrectomy rats (**Figures 2B, C**). Thus, systolic blood pressure at day 7 in 5/6 nephrectomy rats treated with 0.75 mg/kg/day warfarin alone was 161 ± 2 mm Hg, while systolic blood pressure at day 7 in 5/6 nephrectomy rats treated with 150 mg/kg/day dabigatran alone was 161 ± 4.1 mm Hg. In control 5/6 nephrectomy rats systolic blood pressure at day 7 was 134 ± 3.2 mm Hg.

TM5441 reduces systolic blood pressure in both treated and untreated rats, even though the effect of low doses of TM5441 on systolic blood pressure in rats not treated with anticoagulant is not statistically significant. Thus, 5/6 nephrectomy rats treated with 0.75 mg/kg/day warfarin and 2.5 mg/kg/day TM5441 had systolic blood pressure at day 7 of 152 ± 2 mm Hg (versus 0.75mg/kg/day warfarin alone 161 ± 2 mm Hg). In 5/6 nephrectomy rats treated with 0.75 mg/kg/day warfarin and 10.0 mg/kg/day TM5441, systolic blood pressure at day 7 was 144 ± 2mm Hg (**Figure 2B**), ANOVA p=0.0014. Similarly, in 5/6 nephrectomy rats treated with 150 mg/kg/day dabigatran and 2.5 mg/kg/day TM5441 systolic blood pressure at day 7 was 155 ± 2 mm Hg (versus 150 mg/kg/day dabigatran alone 161 ± 4.1 mm Hg). In 5/6 nephrectomy rats treated with 150 mg/kg/day dabigatran and 10.0 mg/kg/day TM5441, systolic blood pressure at day 7 was 144 ± 1 mm Hg (**Figure 2C**), ANOVA p=0.013. Treatment with TM5441 alone decreased systolic blood pressure in 5/6 nephrectomy rats in a dose-depended manner (**Figure 2A**). Treatment with 5 mg/kg/day and 10 mg/kg/day TM5441 decreased blood pressure at day 7 (131 ± 2.1 mm Hg and 130 ± 3.0 mm Hg respectively versus 134 ± 3.2 mm Hg in control, p<0.05). ANOVA p=0.0009. Treatment with 2.5 mg/kg/day TM5441 did not significantly change systolic blood pressure in 5/6 nephrectomy rats at day 7 (132 ± 2.1 mm Hg versus 134 ± 3.2 mm Hg, p=0.2154).

TM5441 reduces diastolic BP in mice treated with warfarin and untreated mice but not in mice treated with dabigatran. Treatment with TM5441 alone in any dose (2.5, 5.0, or 10.0 mg/kd/day) resulted in a significant decrease of diastolic blood pressure (**Figure 2D**), ANOVA p<0.0001. Treatment with 0.75 mg/kg/day warfarin increased diastolic blood pressure in 5/6 nephrectomy rats (**Figure 2E**). Combined treatment with 0.75 mg/kg/day warfarin and TM5441 (2.5, 5.0 and 10.0 mg/kg/day) ameliorated this warfarin-induced increase in diastolic blood pressure in a dose-dependent manner (**Figure 2E**), ANOVA p=0.0084. Treatment with 150 mg/kg/day dabigatran increased diastolic blood pressure in 5/6 nephrectomy rats (**Figure 2F**). Regardless of the dose (2.5, 5.0, or 10.0 mg/kd/day), TM5441 did not affect this dabigatran-induced increase in diastolic blood pressure (**Figure 2F**), ANOVA p=0.1157.

### Effects of PAI-1 inhibitor TM5441 on serum creatinine levels and hematuria in 5/6 nephrectomy rats

**Figure 3:**
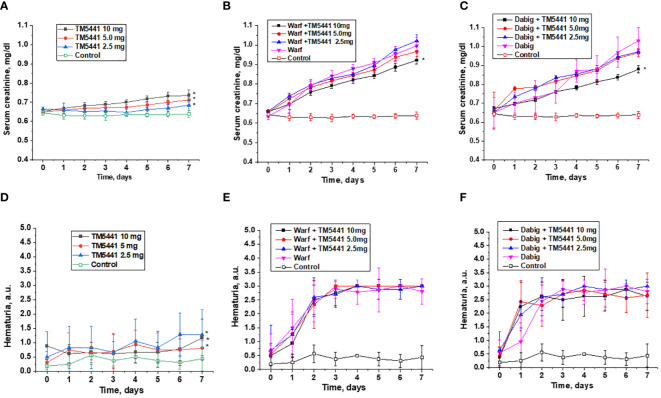
Changes in serum creatinine and hematuria in 5/6 nephrectomy rats treated with warfarin, dabigatran and TM5441. **(A)** changes in serum creatinine in 5/6 nephrectomy rats treated with TM5441 2.5 mg/kg/day, 5.0 mg/kg/day and 10 mg/kg/day. Vehicle treated 5/6 nephrectomy rats were used as control. * - p<0.05 as compared to control. **(B)** changes in serum creatinine in 5/6 nephrectomy rats treated with 0.75 mg/kg/day warfarin (warf) and TM5441 2.5 mg/kg/day, 5.0 mg/kg/day and 10 mg/kg/day. Vehicle treated 5/6 nephrectomy rats were used as control. * - p<0.05 as compared to warfarin alone. **(C)** changes in serum creatinine in 5/6 nephrectomy rats treated with 150 mg/kg/day dabigatran (dab) and TM5441 2.5 mg/kg/day, 5.0 mg/kg/day and 10 mg/kg/day. Vehicle treated 5/6 nephrectomy rats were used as control. * - p<0.05 as compared to dabigatran alone. **(D)** changes in hematuria in 5/6 nephrectomy rats treated with TM5441 2.5 mg/kg/day, 5.0 mg/kg/day and 10 mg/kg/day. Vehicle treated 5/6 nephrectomy rats were used as control. * - p<0.05 as compared to control. **(E)** changes in hematuria in 5/6 nephrectomy rats treated with 0.75 mg/kg/day warfarin (warf) and TM5441 2.5 mg/kg/day, 5.0 mg/kg/day and 10 mg/kg/day. Vehicle treated 5/6 nephrectomy rats were used as control. * - p<0.05 as compared to warfarin alone. **(F)** changes in hematuria in 5/6 nephrectomy rats treated with 150 mg/kg/day dabigatran (dab) and TM5441 2.5 mg/kg/day, 5.0 mg/kg/day and 10 mg/kg/day. Vehicle treated 5/6 nephrectomy rats were used as control. * - p<0.05 as compared to dabigatran alone. Hematuria was evaluated by dipsticks and graded by a semiquantitative scale 0–3, where score 0 is absent, 1+ is trace, 2+ is moderate, and 3+ is large.

Treatment with TM5441 alone resulted in an increase in serum creatinine in 5/6 nephrectomy rats in a dose-dependent manner (**Figure 3A**). Serum creatinine did not change in control 5/6 nephrectomy rats treated with vehicle for the time of observation (**Figure 3A**).

Treatment with warfarin or dabigatran resulted in a rapid increase in serum creatinine in 5/6 nephrectomy rats (**Figures 3B, C**). Treatment with TM5441 ameliorated this anticoagulant-induced increase in serum creatinine in a dose-dependent manner. In both anticoagulants, treatment with 10 mg/kg/day TM5441 significantly affected warfarin and dabigatran – induced increases in serum creatinine. Lower doses of TM5441 also ameliorated the increase in serum creatinine associated with anticoagulation, but this effect was not statistically significant (**Figures 3B, C**).

TM5441 alone increased hematuria in 5/6 nephrectomy rats in a dose-depended manner, p<0.0001 (**Figure 3D**). Hematuria increased in 5/6 nephrectomy rats treated with either warfarin or dabigatran, but this increase was larger than that in 5/6 nephrectomy rats treated with TM5441 alone (**Figures 3E, F**). Treatment with TM5441 did not affect this anticoagulant-associated increase in hematuria for either warfarin or dabigatran (**Figures 3E, F**).

Histologically, there were no changes in the kidney morphology in animals treated with anticoagulants alone or in combination with TM5441. All 5/6 nephrectomy rats treated with warfarin (0.75 mg/kg/day) or dabigatran (150 mg/kg/day) with or without TM5441, had acute tubular epithelial cell injury and red blood cell casts in the tubules, regardless of the dose of TM5441 (2.5, 5.0, or 10.0 mg/kd/day). Control 5/6 nephrectomy rats exhibited mild interstitial fibrosis without RBC casts in the tubules.

### Effects of TM5441 on the reactive oxygen species (ROS) in the kidney in 5/6 nephrectomy rats

**Figure 4:**
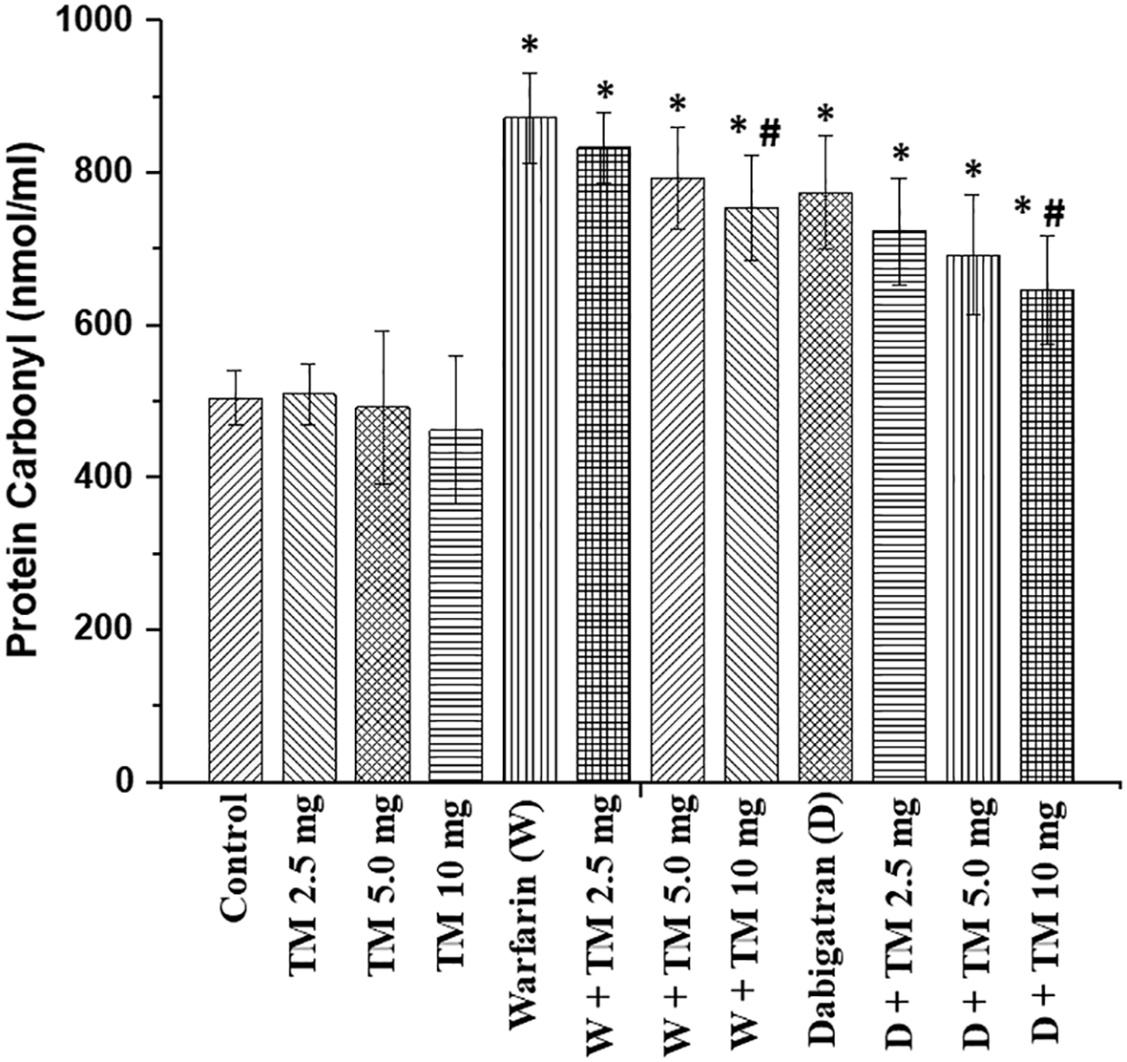
Reactive oxygen species in the kidney in in 5/6 nephrectomy rats treated with warfarin, dabigatran and TM5441. Reactive oxygen species were measured in the renal cortex in the kidney obtained from 5/6 nephrectomy rats treated with 0.75 mg/kg/day warfarin (W), 150 mg/kg/day dabigatran (D) and TM5441 (TM) 2.5 mg/kg/day, 5.0 mg/kg/day and 10 mg/kg/day. Vehicle treated 5/6 nephrectomy rats were used as control. * - p<0.05 as compared to control. # - p<0.05 as compared to anticoagulant alone.

Treatment with dabigatran and warfarin increased ROS in the kidney in 5/6 nephrectomy rats (**Figure 4**). TM5441 alone did not change ROS in the kidneys in 5/6 nephrectomy rats in any dose (2.5, 5.0, or 10.0 mg/kd/day). TM5441 in a dose-dependent manner decreased ROS in the kidneys in 5/6 nephrectomy rats treated with 0.75 mg/kg/day warfarin and TM5441 as compared to 5/6 nephrectomy rats treated with 0.75 mg/kg/day warfarin alone, Thus, the TM5441 10 mg/kg/day reduced warfarin-associated increase inn ROS in the kidney (872 ± 59 nmol/mg vs 755 ± 69 nmol/mg, respectively, p=0.0015). A similar trend was seen in 5/6 nephrectomy rats treated with 150 mg/kg/day dabigatran and TM5441. Hence, 5/6 nephrectomy rats treated with 150 mg/kg/day dabigatran and 10 mg/kg/day TM5441 had significantly lower ROS in the kidney as compared to 5/6 nephrectomy rats treated with 150 mg/kg/day dabigatran alone (774 ± 75 nmol/mg versus 646 ± 72 nmol/mg, respectively, p=0.013), (**Figure 4**).

## Discussion

To the best of our knowledge, this is the first data that describes the role of PAI-1 in the GFB permeability for RBC.

The GFB prevents filtration to the urine of blood cells and large molecules. It is composed from the glomerular capillary endothelial cells, the glomerular basement membrane and the podocytes. In normal physiologic condition, no RBC can cross the GFB into the Bowmans space and further to the proximal tubules. However, in many diseases, the GFB is compromised, and there is glomerular hematuria. ARN is characterized by increased hematuria, formation of RBC casts in the tubules with acute tubular epithelial cell injury/necrosis (ATN), and an increase in serum creatinine. The mechanisms of this increased hematuria in patients on anticoagulation nephropathy are not elucidated, but endothelial cell injury in rats treated with warfarin has been shown by electron microscopy (18).

Our study demonstrated that TM5441 in a dose-dependent manner ameliorates anticoagulant-induced increase in serum creatinine. This was true for both anticoagulant classes: vitamin K antagonist warfarin and direct thrombin inhibitor dabigatran (**Figures 3B, C**). It has been earlier demonstrated that TM55441 alone increased serum creatinine in mice, likely by affecting tubular epithelial cells (19). We also observed the similar phenomenon in our study (**Figure 3A**). TM5441– associated increase in serum creatinine in 5/6NE rats is not accompanied by a ROS increase in the kidney (**Figure 4**). Therefore, other mechanism may play a role in this phenomenon. Unfortunately, acute effects of TM5441 were studied mostly on the liver function, not in the kidney (20). It is theoretically possible that TM5441 affects tubular epithelial cells either directly or by metabolic products (20), changing creatinine excretion/reabsorption. Our data demonstrates that anticoagulant-induced hematuria did not significantly change by addition of TM5441 to the treatment, also, as well as no histological changes were observed in the kidney in 5/6 nephrectomy rats treated with an anticoagulant alone or with combination with TM5441. Even though this data appears to be discordant to the decrease in serum creatinine in 5/6 nephrectomy rats treated with anticoagulants and TM5441, one should take in the account that the quantitation of hematuria by dipstick is not perfect. The number of RBC in the urine would provide better information about the true glomerular hematuria, but such monitoring is difficult to perform in rats even in metabolic cages due to contamination of the urine and degradation of RBC. Ideally, catheterization of the urinary bladder and analysis of middle-portion of the urine should be performed, but it is not feasible in chronic animal studies on small rodents.

Some of the effects of TM5441 could be explained by the reduction in the systemic blood pressure. Both anticoagulants elevated blood pressure in 5/6 nephrectomy rats, as we had reported earlier (7). Theoretically, this should be accompanied by a reduction in the GFR and decreasing hematuria, but our earlier studies demonstrated that GFR-modifying drugs did not affect the severity of ARN in 5/6 nephrectomy rats (8). Therefore, other mechanisms are involved in the serum creatinine decrease, such as the reduction in the oxidative stress in the kidney (**Figure 4**). Our previous studies showed that oxidative stress in the kidney is increased in animals with ARN. ROS inhibition by N-acetylcysteine (NAC) prevents increase in serum creatinine but not glomerular hematuria in 5/6 nephrectomy rats treated with warfarin (16). Beneficial effects of NAC on the kidney function and interstitial fibrosis in the kidney in animals with long-standing hematuria were demonstrated in 5/6 nephrectomy mice treated with warfarin and NAC (21).

PAI-1 is one of the physiological inhibitors of tissue plasminogen activator (t-PA) and urokinase-like plasminogen activator (u-PA). These two enzymes activate the conversion of plasminogen to plasmin. PAI-1 is produced by multiple sources, including endothelial cells, vascular smooth muscle cells, liver, platelets, and podocytes (22–24). It has been demonstrated that ROS upregulate PAI-1 in the kidney by amplification of TGF-beta-1 signaling (25). Inhibition of PAI-1 by TM5441 prevented kidney injury in diabetes in a murine model (19). Long-term effects of PAI-1 on kidney function with the histologic correlate of increased interstitial fibrosis have been demonstrated. These effects are mediated via increased production of TGF-beta-1, decreased plasmin activity, and increased ROS in the kidney (26, 27). On the glomerular level, selective inactivation of PAI-1 in endothelial cells protects glomeruli from senescence and podocyte loss in aging mice (28). It has been suggested that expression of PAI-1 in the glomeruli is associated with glomerular injury, inflammation, and podocyte damage (13). In the podocyte-specific injury model of NEF25/LMB2 mice, there was an increase in PAI-1 in the glomeruli prior to development of thrombosis and proteinuria. Treatment with PAI-1 inhibitor was protective against proteinuria and preserved the number of podocytes in these mice (12). In rats, radiation injury markedly increased PAI-1 mRNA expression at the sites of glomerular injury (29). Upregulation of PAI-1 mRNA was seen in the kidney in patients with crescentic glomerulonephritis (30). *In vitro* studies demonstrated an increased PAI-1 production by cultured human glomerular epithelial cells when they were stimulated by thrombin (31). Delayed branching formation was noted in an *in vitro* model of angiogenesis when aortic rings were treated with PAI-1 (32). Aortic rings obtained from PAI-1 knockout mice had more branching points as compared to the aortic rings obtained from wild type mice (33). These data suggest that PAI-1 injures endothelial cells and this may be responsible for the glomerular filtration barrier injury.

In conclusion, out data demonstrates that PAI-1 inhibitor TM5441 has beneficial effects in 5/6 nephrectomy model of anticoagulant-related nephropathy in rats. TM5441 ameliorates anticoagulant-induced increase in serum creatinine. While no significant effect on hematuria was seen, there was reduction in the ROS in the kidney, which lead to the serum creatinine decrease. Our findings indicate that the inhibition of PAI-1 does not have beneficial effects on the etiology of ARN, such as glomerular hematuria, but ameliorates clinical symptoms, including elevation in serum creatinine.

## Data availability statement

The raw data supporting the conclusions of this article will be made available by the authors, without undue reservation.

## Ethics statement

The animal study was approved by the Institutional Animal Care and Use Committees (IACUC) at the Ohio State University. The study was conducted in accordance with the local legislation and institutional requirements.

## Author contributions

AM: Formal analysis, Validation, Writing – original draft, Data curation, Investigation, Methodology. MX: Data curation, Formal analysis, Investigation, Validation, Writing – original draft, Visualization. LB: Formal analysis, Validation, Visualization, Writing – review & editing. AD: Formal analysis, Visualization, Writing – review & editing, Supervision. AS: Visualization, Methodology, Validation, Writing – original draft. SP: Validation, Visualization, Formal analysis, Writing – review & editing. II: Formal analysis, Validation, Visualization, Writing – review & editing. GM: Formal analysis, Visualization, Writing – review & editing. SB: Formal analysis, Writing – review & editing, Conceptualization, Funding acquisition, Supervision, Validation, Writing – original draft.
